# CSF neurochemical profile and cognitive changes in Parkinson’s disease with mild cognitive impairment

**DOI:** 10.1038/s41531-023-00509-w

**Published:** 2023-04-24

**Authors:** Federico Paolini Paoletti, Lorenzo Gaetani, Giovanni Bellomo, Elena Chipi, Nicola Salvadori, Chiara Montanucci, Andrea Mancini, Marta Filidei, Pasquale Nigro, Simone Simoni, Nicola Tambasco, Massimiliano Di Filippo, Lucilla Parnetti

**Affiliations:** 1 Section of Neurology, Department of Medicine and Surgery, University of Perugia, Perugia, Italy; 2 Laboratory of Clinical Neurochemistry, Section of Neurology, Department of Medicine and Surgery, University of Perugia, Perugia, Italy

**Keywords:** Prognostic markers, Parkinson's disease

## Abstract

Pathophysiological substrate(s) and progression of Parkinson’s disease (PD) with mild cognitive impairment (PD-MCI) are still matter of debate. Baseline cerebrospinal fluid (CSF) neurochemical profile and cognitive changes after 2 years were investigated in a retrospective series of PD-MCI (*n =* 48), cognitively normal PD (PD-CN, *n* = 40), prodromal Alzheimer’s disease (MCI-AD, *n* = 25) and cognitively healthy individuals with other neurological diseases (OND, *n* = 44). CSF biomarkers reflecting amyloidosis (Aβ42/40 ratio, sAPPα, sAPPβ), tauopathy (p-tau), neurodegeneration (t-tau, NfL, p-NfH), synaptic damage (α-syn, neurogranin) and glial activation (sTREM2, YKL-40) were measured. The great majority (88%) of PD-MCI patients was A-/T-/N-. Among all biomarkers considered, only NfL/p-NfH ratio was significantly higher in PD-MCI vs. PD-CN (*p* = 0.02). After 2 years, one-third of PD-MCI patients worsened; such worsening was associated with higher baseline levels of NfL, p-tau, and sTREM2. PD-MCI is a heterogeneous entity requiring further investigations on larger, longitudinal cohorts with neuropathological verification.

## Introduction

Cognitive dysfunctions are frequently reported in Parkinson’s disease (PD), even in newly diagnosed patients, potentially evolving into dementia^1^. Following the paradigm of Alzheimer’s disease (AD) clinical continuum^2^, the concept of mild cognitive impairment (MCI) as a prodromal stage of dementia has been also applied to PD^3,4^. In a recent meta-analysis, Parkinson’s disease with mild cognitive impairment (PD-MCI) has shown variable clinical presentations and progression to dementia^5^.

Different pathophysiological mechanisms are potentially involved in PD-MCI^6^. Although neuropathological data devoted to this specific entity are scanty and carried out in small series, Lewy body pathology, Alzheimer pathology, and cerebral amyloid angiopathy seem to be related to PD-MCI clinical phenotypes^7,8^.

The difficulties in identifying the neurobiological substrate(s) represent a matter of debate about the validity of the MCI construct in the clinical management of PD patients^9^. To give a contribution in this field, we retrospectively analyzed a cohort of patients with PD categorized as PD-MCI^3^ and as cognitively normal (PD-CN), for whom cerebrospinal fluid (CSF) samples collected at the diagnostic work-up were available. As a control group, we considered cognitively healthy individuals who underwent CSF analysis for other minor neurological disorders (OND). We also included, as a contrast group, patients with MCI due to AD (MCI-AD), who have, by definition, a typical CSF neurochemical profile and the highest risk of progression to dementia.

In these groups we measured a large panel of CSF biomarkers reflecting different pathophysiological pathways including amyloidosis (soluble amyloid precursor protein α and β, sAPPα,β; β-amyloid 1–42/1–40 ratio, Aβ42/Aβ40), tauopathy (181-phosphorylated tau, p-tau), amyloid-dependent neurodegeneration (total-tau, t-tau), amyloid-independent neurodegeneration (neurofilament light chain, NfL; phosphorylated neurofilament heavy chain, p-NfH), synaptic damage (total α-synuclein, α-syn; neurogranin, Ng), and glial activation (soluble triggering receptor expressed on myeloid cells 2, sTREM2; chitinase-3-like protein 1, YKL-40). In PD-CN and PD-MCI patients, we also verified the longitudinal changes in cognitive functioning by considering screening tests after 2 years.

The aims of our study were: (i) to investigate whether a well-characterized cohort of PD-MCI patients, compared to PD-CN individuals, show a differential neurochemical profile by measuring a large panel of CSF putative biomarkers reflecting different pathophysiological pathways; (ii) to verify changes in cognitive performances after 2 years, also investigating the potential predictive role of CSF biomarkers on cognitive outcome.

## Results

### Demographic and clinical features

We included 40 PD-CN, 48 PD-MCI, 25 MCI-AD, and 44 OND patients. The details of the main demographic and clinical features of each diagnostic group are summarized in Table 1. At baseline, the great majority of patients were drug-naïve (80% of PD-CN patients and 75% of PD-MCI patients). As expected, PD-MCI patients showed lower Montreal Cognitive Assessment (MoCA) and Mini-Mental State Examination (MMSE) scores, and higher Unified Parkinson’s Disease Rating Scale-part III (UPDRS-III) and Hoehn and Yahr (H&Y) scores, as compared to PD-CN individuals. Details referring to the baseline neuropsychological evaluation in the two diagnostic groups are reported in Table 2.

**Table 1:** Demographic and clinical features in each diagnostic group.

	PD-CN	PD-MCI	MCI-AD	OND	PD-MCI vs. PD-CN
*Baseline*					
*n*	40	48	25	44	–
Sex (F/M)	16/24	22/26	14/11	23/21	–
Age (y)	64 ± 7	68 ± 6	72 ± 5	64 ± 14	–
Disease duration (y)	1.7 ± 1.1	1.9 ± 1.5	1.7 ± 0.6	–	–
H&Y	1.9 ± 0.7	2.1 ± 0.4	–	–	0.0036
UPDRS-III baseline	23.8 ± 10.8	29.2 ± 8.2	–	–	0.0048
MMSE	28.3 ± 1.5	26.0 ± 2.4	21.9 ± 3.3	28.8 ± 0.8	<0.0001
MoCA	25.1 ± 3.1	18.9 ± 3.9	–	–	<0.0001
Patients on dopaminergic treatment	8/40	12/48	–	–	–
LEDD	74 ± 173	107 ± 196	–	–	–
*2-year follow-up*					
*n*	28	37	25	35	
UPDRS-III score change	−6.9 ± 16.6	−2.8 ± 8.2	–	–	–
MMSE score change	−0.1 ± 3.0	−1.0 ± 3.1	−3.1 ± 3.4	−0.2 ± 1.0	–
MoCA score change	0.2 ± 4.3	−1.0 ± 4.2	–	–	0.035

Data are expressed as mean ± standard deviation. Significance level set to 0.005 to account for multiple testing effects in PD-CN vs. PD-MCI comparison. Non-significant *p*-values are not shown.

*H&Y* Hoehn and Yahr stage, *LEDD* L-dopa Equivalent Daily Dosage, *MMSE* Mini-Mental State Examination, *MoCA* Montreal Cognitive Assessment, *MCI-AD* mild cognitive impairment due to Alzheimer’s disease, *OND* healthy cognitive patients with other neurological disorders, *PD-CN* cognitively normal Parkinson’s disease, *PD-MCI* Parkinson’s disease with mild cognitive impairment, *UPDRS-III* Unified Parkinson’s Disease Rating Scale-part III.

**Table 2:** Baseline cognitive scores at neuropsychological tests in PD-MCI and PD-CN groups.

Neuropsychological tests baseline	PD-MCI (*n* = 48)	PD-CN (*n* = 40)	Significance
*Attention and working memory*			
Trail Making Test-A	76.3 ± 33.5	46.7 ± 22.1	<0.0001
Digit Span Forward	5.4 ± 1.7	6.12 ± 1.1	–
Digit Span Backward	3.9 ± 1.7	4.6 ± 1.1	–
Digit Symbol Substitution Test (WAIS-IV)	22.6 ± 8.4	39.7 ± 10.0	<0.0001
*Executive functions*			
Trail Making Test-B	198.7 ± 69.2	125.5 ± 61.0	0.0023
Letter Fluency	24.3 ± 11.7	36.8 ± 10.5	<0.0001
*Language*			
Semantic fluency	29.6 ± 10,0	41.1 ± 8.6	<0.0001
Similarities (WAIS-IV)	13.0 ± 6.2	19.6 ± 5.1	<0.0001
*Memory*			
RAVLT–immediate recall	25.2 ± 7.8	39.4 ± 8.1	<0.0001
RAVLT–delayed recall	4.2 ± 2.3	8.2 ± 2.0	<0.0001
RAVLT–true recognitions	11.1 ± 3.0	13.5 ± 1.6	0.0024
RAVLT– false positive errors	2.9 ± 4.6	0.7 ± 1.2	–
Logical Memory (WMS-IV) - Story recall -	7.8 ± 2.9	13.9 ± 4.8	<0.0001
*Visuospatial functions*			
Copy Drawing Test (MDB)	8.3 ± 2.0	10.3 ± 1.3	<0.0001
Copy Drawing Test with Landmarks (MDB)	63.7 ± 7.2	66.7 ± 4.0	–
Clock Drawing Test	4.3 ± 2.8	0.6 ± 2.1	<0.0001

Data are expressed as mean ± standard deviation.

The significance level was set to 0.0026 to account for multiple testing effects, non-significant *p*-values are not shown.

*MDB* Mental Deterioration Battery, *RAVLT* Rey’s Auditory Verbal Learning Test, *WAIS-IV* Wechsler Adult Intelligence Scale–Fourth Edition, *WMS-IV* Wechsler Memory Scale-Fourth Edition.

At the comprehensive clinical evaluation carried out after two years, the diagnosis of PD was confirmed in all patients. Out of 88 PD patients, neuropsychological assessment at follow-up was available for 65 (28 PD-CN and 37 PD-MCI). Cognitive worsening (loss of at least 2 points at both MMSE and MoCA) was observed in 30% of PD-MCI patients, as opposed to 43% of PD-MCI patients with unchanged scores. None of PD-CN individuals worsened both at MMSE and MoCA (Table 3). In Supplementary Table 1 follow-up scores obtained in all neuropsychological tests are reported.

**Table 3:** Prevalences of PD-MCI and PD-NC patients who remained cognitively stable or worsened (at least 2 points loss) at MMSE, MoCA and both MMSE and MoCA after 2 years.

	PD-MCI (*n* = 37)	PD-CN (*n* = 28)
*MMSE*		
stable	62%	82%
2 points loss	38%	18%
*MoCA*		
stable	51%	93%
2 points loss	49%	7%
*MMSE and MoCA*		
stable	43%	75%
2 points loss	30%	—

*MoCA* Montreal Cognitive Assessment, *MMSE* Mini-Mental State Examination.

### CSF biomarkers

**Fig. 1:**
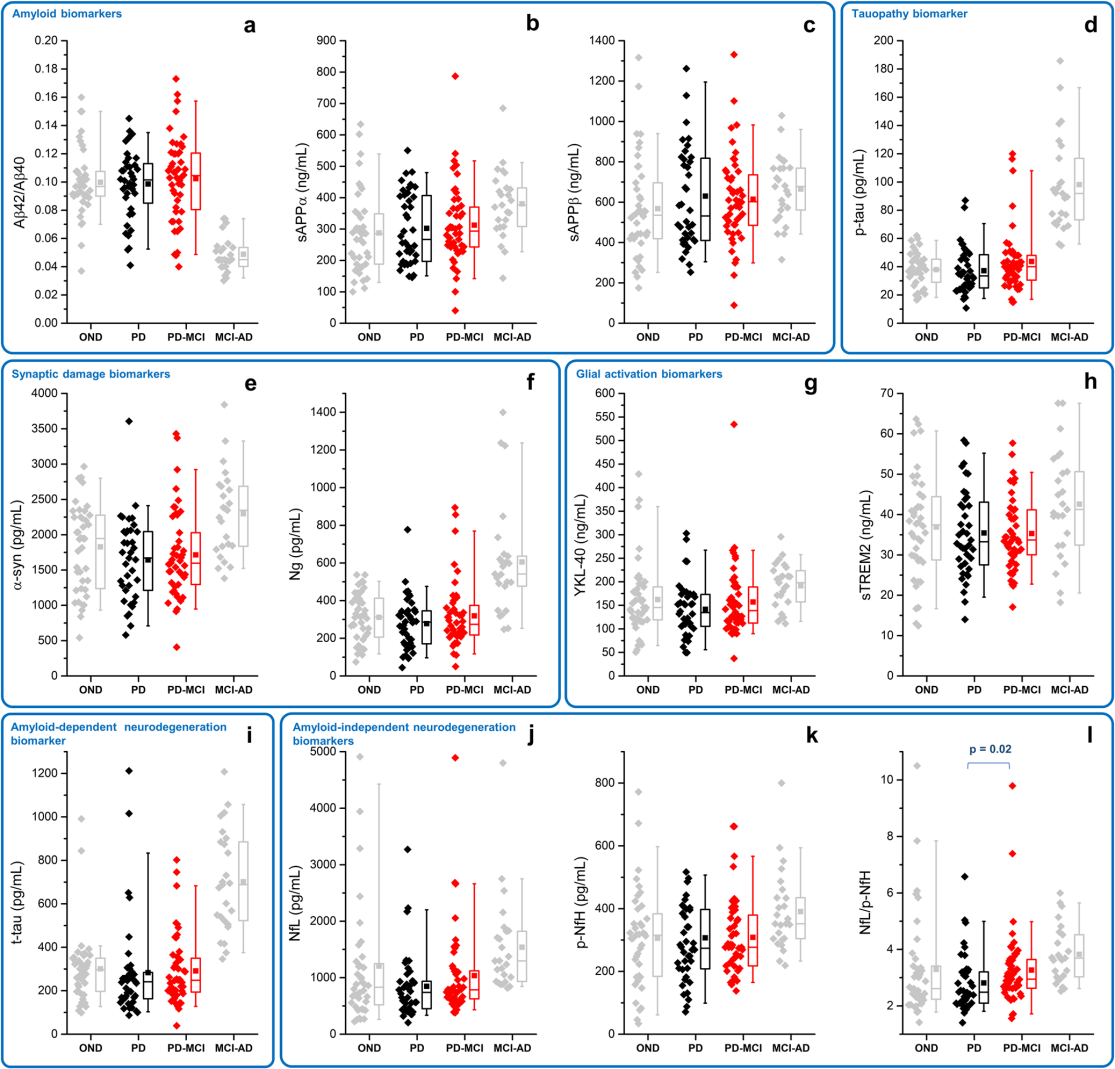
Box plots relative to biomarkers measured in PD-MCI, PD-CN, MCI-AD and OND. **a**–**c** amyloid biomarkers: Aβ42/40, sAPPα, sAPPβ. **d** tauopathy biomarker: p-tau. **e**, **f** synaptic damage biomarkers: α-syn, Ng. **g**, **h** glial activation biomarkers: YKL-40, sTREM2. **i** amyloid-dependent neurodegeneration biomarker: t-tau. **j–l** amyloid-independent neurodegeneration biomarkers: NfL, p-NfH, NfL/p-NfH. Boxplots summarize the distribution of the data, where the box represents the interquartile range, the horizontal line inside the box represents the median, and the filled square within the box represents the mean. The whiskers extend to the minimum and maximum values within 90% of the data range. P-values have been corrected for multiple group comparisons. MCI-AD mild cognitive impairment due to Alzheimer’s disease, OND healthy cognitive patients with other neurological disorders, PD-CN cognitively normal Parkinson’s disease, PD-MCI Parkinson’s disease with mild cognitive impairment. Aβ42/Aβ40 β-amyloid 1-42/1-40 ratio, NfL neurofilament light chain, Ng neurogranin, p-NfH phosphorylated neurofilament heavy chain, p-tau phosphorylated-tau, sAPPα soluble amyloid precursor protein α, sAPPβ soluble amyloid precursor protein β, sTREM2 soluble triggering receptor expressed on myeloid cells 2, α-syn total α-synuclein, t-tau total tau, YKL-40 chitinase-3-like protein 1.

Biomarker’s concentrations in PD-CN, PD-MCI, MCI-AD, and OND are plotted in Fig. 1. Mean values of all CSF biomarkers in the four groups are reported in Supplementary Table 2.

### AD biomarkers

CSF Aβ42/40 ratio, p-tau, t-tau, sAPPα, and sAPPβ levels did not differ among PD-MCI, PD-CN, and OND. With respect to A/T/(N) profile, 3 (6%) out of PD-MCI patients and 2 (5%) out of PD-CN patients showed a CSF AD-like profile.

### Amyloid-independent neurodegeneration biomarkers

CSF NfL showed a slight trend toward an increase in PD-MCI compared to PD-CN (*p* = 0.09), while CSF p-NfH levels were similar in PD-MCI, PD-CN, and OND. NfL/p-NfH ratio was significantly higher in PD-MCI vs. PD-CN (*p* = 0.02). This difference was still statistically significant after adjusting for age, sex, and disease duration (*p* adjusted = 0.036).

CSF NfL was significantly higher in MCI-AD compared to all other groups (*p* = 0.0001). CSF p-NfH and NfL/p-NfH ratios were significantly increased in MCI-AD with respect to PD-MCI and PD-CN.

### Biomarkers of synaptic damage

CSF α-syn and Ng levels were similar in PD-MCI, PD-CN, and OND patients. As expected, they were higher in MCI-AD vs. the other groups.

### Biomarkers of glial activation

CSF YKL-40 and sTREM2 did not differ among PD-MCI, PD-CN, and OND groups. Both were increased in MCI-AD compared to all other groups.

### Correlations among CSF biomarkers

Spearman’s correlation coefficients among CSF biomarkers are reported in two heatmaps (Supplementary Figure). Ng and α-syn concentrations strongly correlated with each other; they correlated with t-tau in PD-MCI and PD-CN, with p-tau in PD-MCI, and with YKL-40 in PD-CN. In all groups, NfL and p-NfH levels, as well as sAPPα and sAPPβ, were strongly correlated.

### CSF biomarkers association with clinical measures

CSF biomarkers did not show any correlation with baseline UPDRS-III, Levodopa Equivalent Daily Dose (LEDD), MMSE, and MoCA scores in PD groups.

**Fig. 2:**
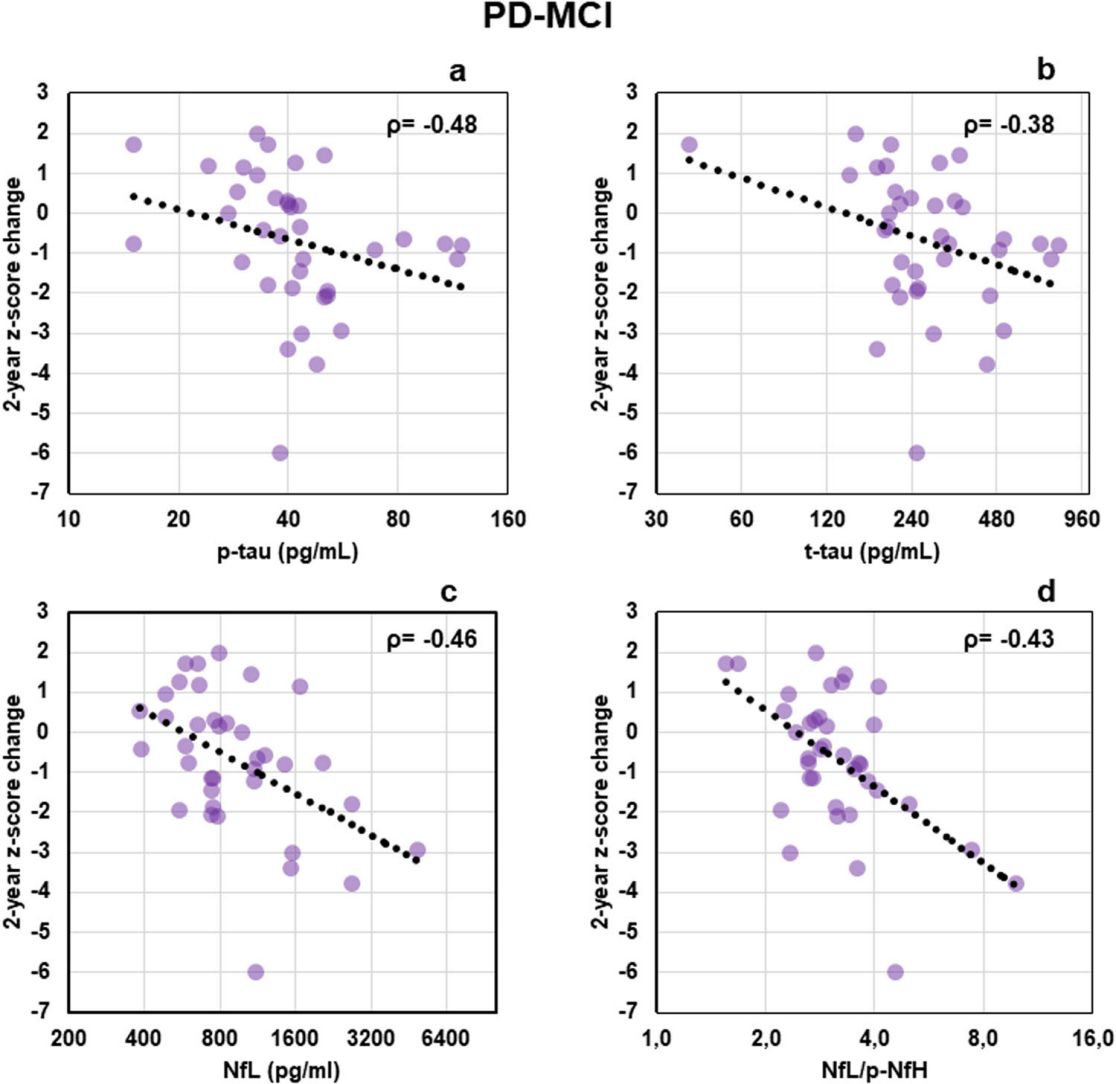
Scatter plots of correlations between biomarkers and *Z*-score change of cognitive tests. **a** p-tau, **b** t-tau, **c** NfL, **d** NfL/p-NfH. The reported correlations produced, in the different groups, Spearman correlation coefficients significantly different from 0 after FDR correction and *p*-values < 0.05 after a nonparametric regression that considered age, sex, and disease duration as covariates. In order to allow a better visual interpretation of the Spearman correlation, biomarker values are shown on a log2-scale, a log fit was also added for visual purposes. NfL neurofilament light chain, p-NfH phosphorylated neurofilament heavy chain, p-tau phosphorylated tau protein, t-tau total tau protein.

In Fig. 2 the associations between CSF biomarkers and *Z*-score changes at neuropsychological tests are reported. In PD-MCI group, *Z*-score change was significantly associated with p-tau (*ρ* = −0.48, FDR-adjusted *p* = 0.01), t-tau (*ρ* = −0.38, FDR-adjusted *p* = 0.02), NfL (*ρ* = −0.46, FDR-adjusted *p* = 0.02), and NfL/p-NfH (*ρ* = −0.43, FDR-adjusted *p* = 0.03).

**Fig. 3:**
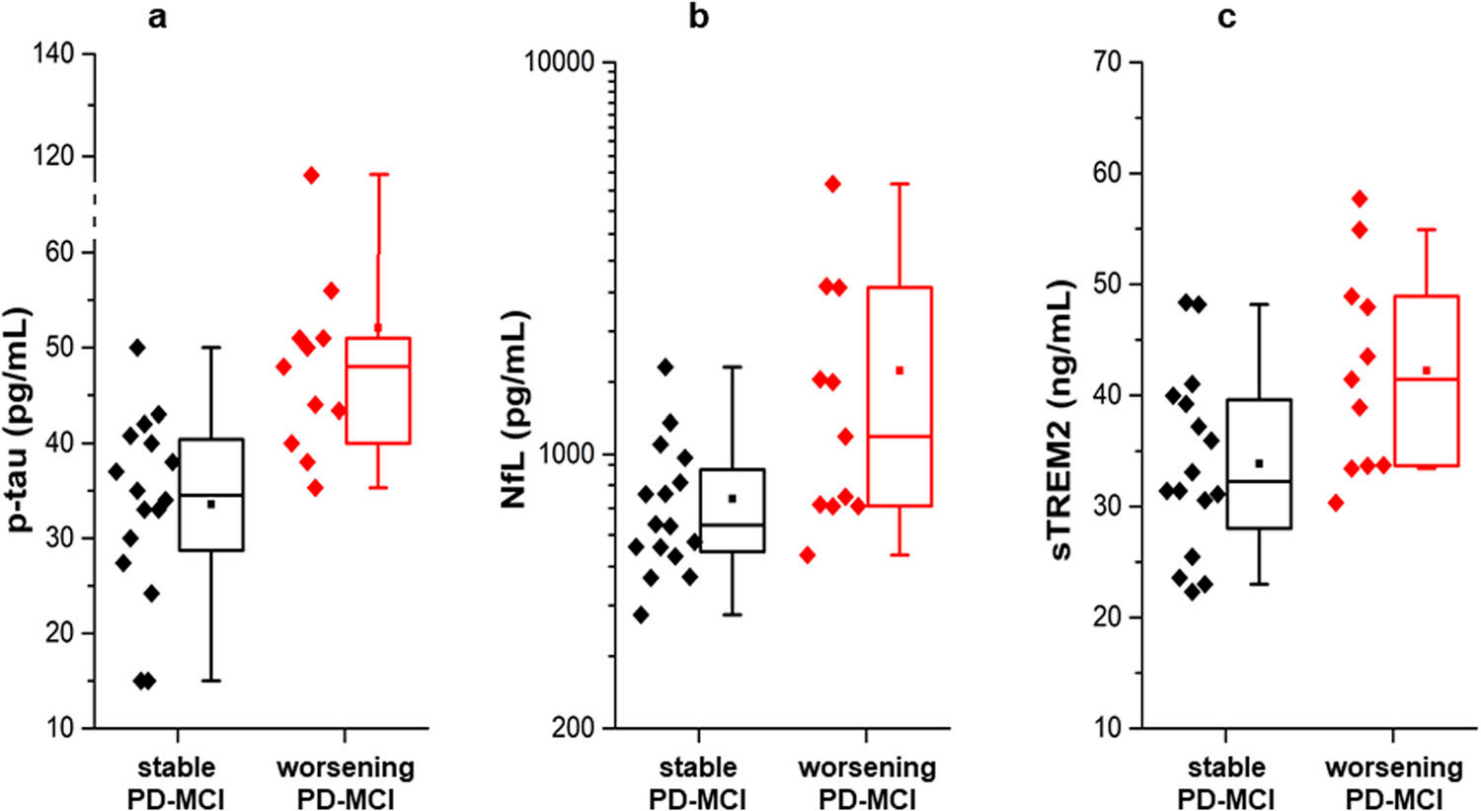
Box plots showing differences in CSF biomarkers levels between PD-MCI patients showing at least 2 point-loss at both MMSE and MoCA (worsening PD-MCI, red) and PD-MCI without cognitive worsening (stable PD-MCI, black). **a** difference in CSF p-tau between worsening PD-MCI and stable PD-MCI. **b** difference in CSF NfL between worsening PD-MCI and stable PD-MCI. **c** difference in CSF sTREM2 between worsening PD-MCI and stable PD-MCI. In boxplots, box height represents the interquartile range, the square represents the mean, the horizontal line represents the median and whiskers represent the 5–95% range. Biomarker values are shown in the log-scale for viewing purposes. NfL neurofilament light chain, p-tau phosphorylated tau protein, sTREM2 soluble triggering receptor expressed on myeloid cells 2.

PD-MCI patients showing cognitive worsening had significantly higher levels of CSF p-Tau, NfL, and sTREM2, when compared to cognitively stable PD-MCI patients (*p* = 0.0017, *p* = 0.042, and *p* = 0.047, respectively, Fig. 3).

## Discussion

In this retrospective study, we wanted to verify if the CSF neurochemical profile, explored by means of a large panel of CSF biomarkers, and the clinical outcome of patients diagnosed as PD-MCI show differential features when compared to PD-CN individuals. To the best of our knowledge, such a large panel of CSF biomarkers has not been investigated before in the specific category of PD-MCI.

In our series, out of all biomarkers assessed, only the NfL/p-NfH ratio showed to be significantly higher in PD-MCI vs. PD-CN. After 2 years, one-third of PD-MCI patients showed a cognitive worsening, associated with baseline higher levels of NfL, p-tau, and sTREM2.

CSF biomarkers reflecting AD pathology are the most investigated biomarkers in PD. According to the A/T/(N) classification^2^, AD pathology is characterized by the presence of amyloidosis (A+) and tauopathy (T+). In CSF, brain amyloidosis is reflected by decreased Aβ42/Aβ40 ratio (A+) and tauopathy by increased p-tau levels (T+). Several post-mortem studies report the possible coexistence of AD pathology in PD brains^7,8^, also suggesting a relationship with the development of dementia in PD^10^. We thus assessed in our cohort CSF A/T/(N) profile, in order to evaluate the possible coexistence of AD pathology. CSF AD-like profile has been reported in 9–28% of Parkinson’s disease with dementia (PDD) series^11,12^. PD-MCI patients may show either normal^13–15^ or altered^16–19^ CSF AD biomarkers. In a previous work^12^, we found that 87% of PD-MCI subjects show a CSF A-/T-/(N-) profile, consistent with the findings of the present study. Previous data indicated that low baseline CSF Aβ42 levels may predict cognitive decline in PD^20–22^. In the present study, p-tau and t-tau seem to better predict cognitive worsening in PD-MCI. The association between CSF tau species and cognitive decline in PD has been previously reported in DATATOP^23^ and BioFINDER^24^ cohorts.

As a measure of axonal degeneration, we investigated CSF NfL, pNfH, and their ratio. Available data consistently show increased levels of NfL in neurodegenerative disorders with worse outcomes, including atypical parkinsonisms as compared to idiopathic PD^25^. Several reports consistently indicate that increased CSF NfL levels at baseline predict cognitive decline in PD patients^14,15,26–28^. Available findings concerning CSF NfL in PD-MCI are inconsistent, showing either increased^15,26,27^ or unchanged^14^ levels vs. cognitively healthy PD. In our cohort, the CSF NfL/p-NfH ratio could show a statistically significant difference between PD-MCI and PD-CN, and CSF NfL was significantly higher in PD-MCI patients showing cognitive worsening with respect to cognitively stable PD-MCI individuals. Additionally, both CSF NfL and NfL/p-NfH were positively associated with *Z*-score change of cognitive tests in PD-MCI, which further strengthens the role of CSF neurofilaments as markers of worse cognitive outcome in this group of patients.

CSF biomarkers assessing synaptic dysfunction, i.e., α-syn and Ng, did not differ between PD-MCI and PD-CN. In previous studies, CSF α-syn has been shown to be decreased in PD patients when compared to controls^29,30^, as a possible consequence of its accumulation within the Lewy bodies. However, data from different meta-analyses consistently reported poor diagnostic accuracy of CSF α-syn in discriminating PD^31,32^. Afterward, CSF α-syn has been found to increase in PD along with disease duration^24,33^, being also related to the clinical outcome (higher the levels, worse the motor outcome)^22^. With respect to the relationship between CSF α-syn levels and cognitive decline in PD, available data are inconsistent^22,34–37^. In a previous report, no difference in CSF α-syn levels was found between PD-MCI and cognitively unimpaired PD^15^. Since there is evidence about the increase of CSF α-syn levels with disease duration^24,33^, the relatively low levels of α-syn found both in PD-MCI and in PD-CN in our series might be also due to the short disease duration of the patients included. CSF α-syn increase has been documented in other neurodegenerative disorders unrelated to α-syn pathology, namely AD^38^, Creutzfeldt–Jakob disease^39^ and progressive supranuclear palsy^40^. Taken together, these findings indicate that CSF α-syn levels unspecifically reflect the ongoing synaptic damage, as already previously proposed^22,41,42^. However, studies specifically assessing CSF α-syn as a biomarker of synaptic damage in the group of PD-MCI, are actually scanty.

In our series, CSF Ng was increased in MCI-AD, in agreement with previous reports^43,44^, being unchanged both in PD-CN and PD-MCI. Previous studies in PD cohorts show either unchanged^44,45^ or decreased levels^46–48^. Ng has been defined as the biomarker of cognition, being specifically related to cognitive functioning not only in AD^44^, but also in normal aging^49^ and in PD^48,50^—the higher the levels, the worse the performance, as a reflection of cortical synaptic damage. Accordingly, in the majority of PD cases, we could justify unchanged CSF Ng levels as a consequence of little/no cortical involvement. With respect to the specific category of PD-MCI, available data are scanty^44^, making necessary further investigations on this entity.

CSF YKL-40 and sTREM2, reflecting astrocytic and microglial activation respectively, were unchanged both in PD-CN and PD-MCI. Increased levels of these inflammatory markers have been reported in PD patients with MoCA scores <26^26^. On the contrary, normal levels of CSF sTREM2 have been recently found in both PD-MCI and PD-CN^51^. In our series, increased baseline levels of sTREM2 were observed in PD-MCI patients showing cognitive worsening after two years. Accordingly, different investigations from the PPMI cohort reported that CSF sTREM2 has a role in predicting cognitive decline in PD^26,52^, being also increased in those patients with positive CSF tau signature^51–53^. All these findings, taken together, seem to suggest that CSF sTREM2 may be a promising predictor of worse cognitive outcome rather than a diagnostic biomarker in PD-MCI.

As a limitation of our work, we should acknowledge the short follow-up available for our cohort when assessing the progression rate of cognitive impairment in PD. This limitation is partially counterbalanced by the clinical homogeneity of the PD cohort and the thorough neuropsychological and CSF characterization.

Overall, in our retrospective cohort, when compared to cognitively unimpaired PD subjects, patients categorized as PD-MCI did not show a differential CSF neurochemical profile. Not all PD-MCI patients showed cognitive worsening after 2 years (although we should acknowledge the relatively short time window of observation), indicating some heterogeneity in this category, which is in line with previous literature demonstrating that up to 30% of PD-MCI patients can even revert to a normal cognition along a follow-up ranging from 3 to 5 years^54,55^. Of interest, baseline CSF p-tau, NfL, and sTREM2 were significantly higher in PD-MCI patients showing cognitive worsening, which suggests the possibility to consider these CSF biomarkers as prognostic factors. Our data highlight the need for further investigations on larger, longitudinal cohorts of PD-MCI patients including neuropathological verification.

## Methods

Among patients referred to our Center in the period 2016–2019, we retrospectively selected a consecutive series of 88 PD patients, 25 MCI-AD patients, and 44 cognitively healthy patients with OND, i.e., mononeuropathy, dizziness, and headache, for whom CSF and clinical follow-up at 2 years could be available. Similar to what was done for Alzheimer’s disease, in our Center CSF analysis is a routine procedure also in PD patients, as approved by our local Ethics Committee (protocol No.19369/08/AV, registry No. 1287/08, date: 9 October 2008). The study was approved by our local Ethics Committee *CER Umbria*. All patients gave their written informed consent for the study participation.

### PD patients

We included only PD patients with satisfactory motor performance, thus functionally independent, with relatively short disease duration, in order to have a quite homogeneous cohort and to avoid the impact of motor impairment on cognitive functioning. Therefore, the following criteria were considered. Inclusion criteria were: (i) diagnosis of clinically established PD, according to the Movement Disorder Society (MDS) criteria^56^, (ii) optimal response to dopaminergic challenge test, (iii) age at lumbar puncture (LP) between 60 and 80 years old, (iv) disease duration since symptoms onset <5 years, (v) early—moderate disease stage (H&Y^57^ < 3), (vi) de novo drug-naïve patients or patients treated with low-middle dosage of dopaminergic drugs, i.e., LEDD^58^ ≤ 600 mg per day. Exclusion criteria included: (i) L-dopa-induced dyskinesia and motor fluctuations, as well as behavioral disturbances, (ii) diagnosis of PDD, (iii) diagnosis of atypical parkinsonian syndromes, (iv) relevant vascular encephalopathy on brain imaging and recent major stroke (<6 months), (v) recent traumatic brain injury (<30 days); (vi) major systemic disorders. At baseline, PD patients underwent a thorough clinical evaluation including motor assessment and neuropsychological testing, as well as brain imaging to rule out other causes. Motor impairment and disease-related disability were assessed by means of UPDRS-III^59^ and H&Y^57^, respectively. Neuropsychological evaluation included MMSE and MoCA as cognitive screening tests, and a comprehensive neuropsychological battery exploring attention and working memory, executive functions, memory, language, and visuospatial functions. Based on neuropsychological evaluation, patients with no cognitive impairment were defined as PD-CN, while PD-MCI patients were defined according to MDS Task Force criteria, level II^3^. After 2 years, cognitive worsening was defined as a loss of at least 2 points in both MMSE and MoCA, based on available evidence from previous literature^60–62^.

### MCI-AD patients

Inclusion criteria were: (i) diagnosis of AD according to the CSF biomarkers profile A+/T+, (ii) score at Clinical Dementia Rating (CDR) scale = 0.5, (iii) age at LP between 60 and 80 years.

### OND patients

Inclusion criteria were: (i) minor neurological diseases for which CSF analysis was performed as part of the diagnostic work-up, (ii) CSF AD core biomarkers in the normal range, (iii) normal neuropsychological evaluation at baseline and at 2-year follow-up, (iv) no vascular encephalopathy, recent major stroke, brain injury and major systemic disorders, (v) no ongoing treatments with anti-inflammatory drugs.

### CSF sampling and analysis

LP was performed according to international guidelines^63^. Briefly, 10–12 mL of CSF were collected in sterile polypropylene tubes and centrifuged at room temperature for 10 min (2000×*g*). Aliquots (0.5 mL) were frozen at −80 °C. All the analyses were carried out by means of commercially available kits. Aβ40, Aβ42, t-tau and p-tau were analyzed using Lumipulse G600-II fully automated chemiluminescent enzyme immunoassay system (Fujirebio Europe, Gent, Belgium). NfL concentrations were determined with NF-light (UmanDiagnostics, Umeå, Sweden), p-NfH with p-NfH (Euroimmun, Lubeck, Germany), Ng with Neurogranin (Trunc P75) (Euroimmun, Lubeck, Germany), t-α-syn with Alpha-Synuclein (Euroimmun, Lubeck, Germany), sAPPα with sAPP-alpha (Tecan, Männedorf, Switzerland), sAPPβ with sAPP-beta wild type (Tecan, Männedorf, Switzerland), sTREM2 with Human TREM2 (Abcam, Cambridge, United Kingdom), and YKL-40 with Human Chitinase 3-like 1 Quantikine (R&D Systems, Minneapolis, MN). All analyses were performed by board-certified laboratory technicians blinded to clinical data. The analyses were performed using one batch of reagents with intra-assay coefficients of variation below 10%.

### Statistical analysis

The data analysis was performed by using OriginPro 9 and R v3.6. Normality of both clinical and biochemical features was assessed by means of the Shapiro–Wilk test. Considering the non-normal distribution of some clinical and biochemical variables, the Wilcoxon–Mann–Whitney sum rank test was used to compare biomarkers distributions among groups. All p-values were corrected for multiple group comparisons^64^. To combine the score changes of both MMSE and MoCA, a *Z*-score change was calculated^65^. Correlations among CSF biomarkers, and among CSF biomarkers and clinical measures (MoCA, MMSE, UPDRS-III and H&Y, LEDD, and *Z*-score change) were calculated by means of Spearman’s (*ρ*) correlation coefficient. When assessing the significance of the correlations between biomarkers and *Z*-score changes at two-year follow-up, *p*-values were adjusted for false-discovery-rate (FDR)^64^. Linear regression was also applied to further assess the significance of the differences observed among PD groups and the significance of the associations between biomarker levels and clinical parameters by assuming age, sex, and disease duration as covariates. Prior to linear regression, data were rank-transformed to account for non-normality. Logistic regression was applied to determine differences in CSF biomarkers levels between PD-MCI with and without cognitive worsening assuming age, sex, and disease duration as covariates.

### Reporting summary

Further information on research design is available in the Nature Research Reporting Summary linked to this article.

## Supplementary information

Supplementary Materials

Reporting Summary

## Data Availability

All relevant data generated during this study are available from the corresponding author on reasonable request.
