# Supplementary material for: CSF neurochemical profile and cognitive changes in Parkinson’s disease with mild cognitive impairment
Source: NPJ Parkinsons Dis. 2023 Apr 24;9:68. doi: 10.1038/s41531-023-00509-w (PMC10126070; doi:10.1038/s41531-023-00509-w)
Supplement: Supplementary file 1 — Supplementary Materials [file 41531_2023_509_MOESM1_ESM.pdf]

SUPPLEMENTARY MATERIALS

Supplementary Table 1

Cognitive scores after 2 years at neuropsychological tests in PD-MCI and PD-CN groups

Neuropsychological Test ***2-YEAR FOLLOW-UP***	**PD-MCI** (n=37)	**PD-CN** (n=28)
Attention and working memory		
Trail Making Test - A	90.6±45.2	61.9±41.2
Digit Span Forward	5.0±0.8	5.7±1.1
Digit Span Backward	2.9±1.6	3.7±1.3
Digit Symbol Substitution Tests (WAIS-IV)	51.0±22.7	52.5±29.4
Executive functions		
Trail Making Test - B	217.8±80.6	153.2±78.1
Letter Fluency	18.3±7.9	28.7±13.3
Language		
Semantic Fluency	28.5±9.5	35.6±11.1
Memory		
RAVLT - immediate recall	24.9±6.7	34.3±11.1
RAVLT - delayed recall	4.3±2.2	6.7±3.4
RAVLT - true recognitions	12.4±2.7	13.2±2.3
RAVLT - false positive errors	3.4±3.6	1.6±2.5
Logical Memory (WMS-IV) - Story recall	10.7±3.3	12.7±4.4
Visuospatial functions		
Copy Drawing Test (MDB)	8.3±1.6	9.3±1.7
Copy Drawing Test with Landmarks (MDB)	59.1±5.0	63.5±5.5
Clock Drawing Test	4.1±3.7	2.5±2.8

Data are expressed as mean ± standard deviation.

Abbreviations. MDB: Mental Deterioration Battery; RAVLT: Rey's Auditory Verbal Learning Test WAIS-IV: Wechsler Adult Intelligence Scale – Fourth Edition; WMS-IV: Wechsler Memory Scale- Fourth Edition

Supplementary Table 2
CSF biomarkers mean values in each diagnostic group

		Mean	SD	Min	Median	Max	IQR
**Aβ42/40**	PD-CN	0.099	0.025	0.041	0.102	0.145	0.028
	PD-MCI	0.102	0.030	0.040	0.105	0.173	0.040
	MCI-AD	0.049	0.012	0.030	0.045	0.074	0.013
	OND	0.101	0.022	0.065	0.097	0.160	0.018
**sAPPα (ng/mL)**	PD-CN	302	113	146	267	550	210
	PD-MCI	313	127	40	293	787	128
	MCI-AD	381	110	144	377	685	123
	OND	294	134	100	285	634	163
**sAPPβ (ng/mL)**	PD-CN	630	280	253	532	1484	408
	PD-MCI	615	223	89	602	1331	251
	MCI-AD	665	166	315	678	1029	207
	OND	575	251	174	535	1316	287
**p-tau (pg/mL)**	PD-CN	37.2	16.5	10.8	33.5	87.0	23.5
	PD-MCI	43.7	22.5	15.0	40.0	120.0	17.5
	MCI-AD	98.2	33.8	55.0	91.9	185.8	43.5
	OND	36.3	14.9	9.8	38	62.0	21.2
**t-tau (pg/mL)**	PD-CN	283	229	87	241	1212	121
	PD-MCI	291	160	39	248	802	156
	MCI-AD	702	236	346	688	1208	362
	OND	284	178	20	270	991	151
**NfL (pg/mL)**	PD-CN	847	588	205	739	3271	486
	PD-MCI	1037	766	380	786	4893	496
	MCI-AD	1540	863	816	1296	4800	890
	OND	1261	1339	142	800	5498	840
**p-NfH (pg/mL)**	PD-CN	307	181	71	274	1161	190
	PD-MCI	308	123	138	277	662	162
	MCI-AD	391	129	219	352	800	131
	OND	380	441	34	315	2726	222
**NfL/p-NfH**	PD-CN	2.82	1.04	1.41	2.49	6.58	1.11
	PD-MCI	3.27	1.36	1.56	2.95	9.79	1.02
	MCI-AD	3.83	1.00	2.53	3.69	6.00	1.49
	OND	4.28	4.1	1.44	2.84	21.2	1.60
**t-α-syn (pg/mL)**	PD-CN	1645	599	580	1670	3607	833
	PD-MCI	1715	623	408	1598	3430	734
	MCI-AD	2299	609	1383	2347	3841	850
	OND	1889	827	541	1943	5336	1115
**Ng (pg/mL)**	PD-CN	278	140	45	286	777	175
	PD-MCI	319	175	50	274	894	157
	MCI-AD	606	296	247	542	1400	160
	OND	325	175	74	312	1095	227
**YKL-40 (ng/mL)**	PD-CN	142	58	50	135	303	68
	PD-MCI	157	77	37	139	534	77
	MCI-AD	192	48	111	198	296	66
	OND	159	80	50	143	429	69
**sTREM2 (ng/mL)**	PD-CN	35	10	14	33	58	15
	PD-MCI	35	9	17	33	57	11
	MCI-AD	42	13	18	41	67	18
	OND	36	13	12	37	63	16

Abbreviations. Aβ42/Aβ40: β-amyloid 1-42/1-40 ratio; IQR: interquartile range; NfL: neurofilament light chain; Ng: neurogranin; p-NfH: phosphorylated neurofilament heavy chain; p-tau: phosphorylated-tau; sAPPα: soluble amyloid precursor protein α; sAPPβ; soluble amyloid precursor protein β; sTREM2: soluble triggering receptor expressed on myeloid cells 2; t-α-syn: total α-synuclein; t-tau: total tau; YKL40: chitinase-3-like protein 1.

Supplementary Figure

(A) Spearman's correlation coefficients among measured biomarkers in PD-MCI and PD-CN. (B) Spearman's correlation coefficients among measured biomarkers in PD-MCI and MCI-AD.

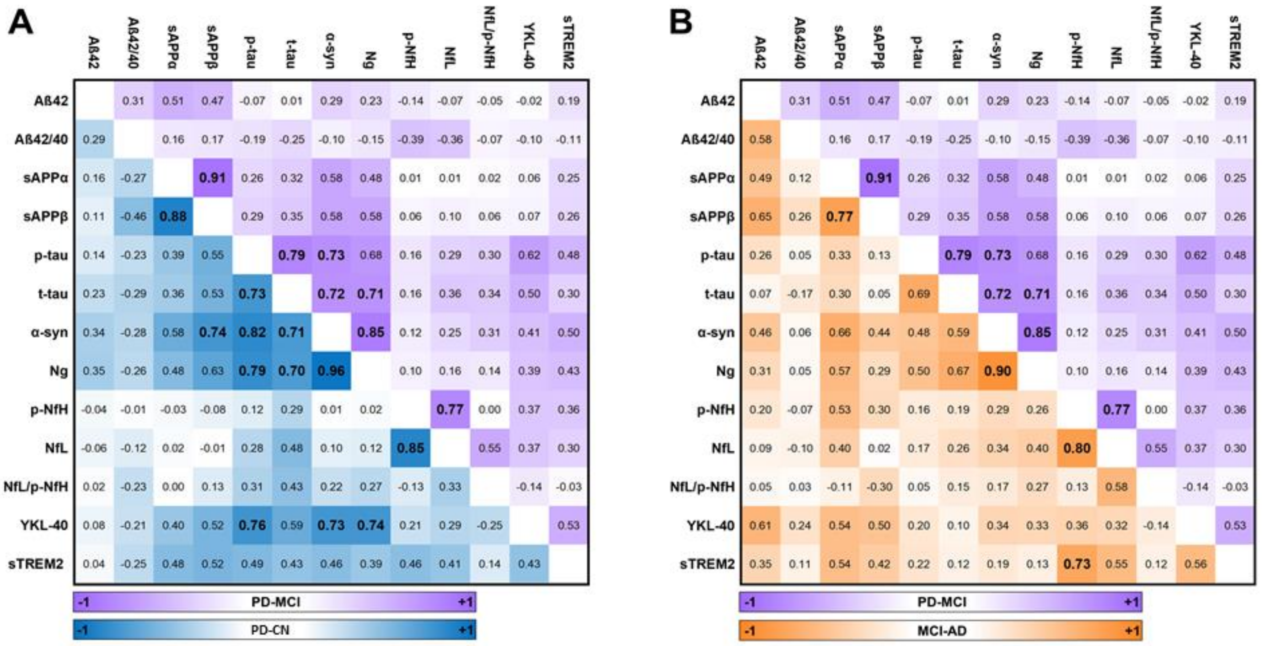

Correlation coefficients higher than 0.7 (strong correlation) are highlighted in bold.

Abbreviations. Aβ42/Aβ40: β-amyloid 1-42/1-40 ratio; NfL: neurofilament light chain; Ng: neurogranin; p-NfH: phosphorylated neurofilament heavy chain; p-tau: phosphorylated-tau; sAPPα: soluble amyloid precursor protein α; sAPPβ; soluble amyloid precursor protein β; sTREM2: soluble triggering receptor expressed on myeloid cells 2; t-α-syn: total α-synuclein; t-tau: total tau; YKL40: chitinase-3-like protein 1.
