# Supplementary material for: CSF neurochemical profile and cognitive changes in Parkinson’s disease with mild cognitive impairment
Source: NPJ Parkinsons Dis. 2023 Apr 24;9:68. doi: 10.1038/s41531-023-00509-w (PMC10126070; doi:10.1038/s41531-023-00509-w)
Supplement: Supplementary file 2 — Reporting Summary [file 41531_2023_509_MOESM2_ESM.pdf]

nature portfolio

Corresponding author(s): Lucilla Parnetti

Last updated by author(s): Apr 4, 2023

# Reporting Summary

Nature Portfolio wishes to improve the reproducibility of the work that we publish. This form provides structure for consistency and transparency in reporting. For further information on Nature Portfolio policies, see our Editorial Policies and the Editorial Policy Checklist.

## Statistics

For all statistical analyses, confirm that the following items are present in the figure legend, table legend, main text, or Methods section.

| n/a | Confirmed | |
|---|---|---|
| ☐ | ☒ | The exact sample size (*n*) for each experimental group/condition, given as a discrete number and unit of measurement |
| ☐ | ☒ | A statement on whether measurements were taken from distinct samples or whether the same sample was measured repeatedly |
| ☐ | ☒ | The statistical test(s) used AND whether they are one- or two-sided<br>*Only common tests should be described solely by name; describe more complex techniques in the Methods section.* |
| ☐ | ☒ | A description of all covariates tested |
| ☐ | ☒ | A description of any assumptions or corrections, such as tests of normality and adjustment for multiple comparisons |
| ☐ | ☒ | A full description of the statistical parameters including central tendency (e.g. means) or other basic estimates (e.g. regression coefficient) AND variation (e.g. standard deviation) or associated estimates of uncertainty (e.g. confidence intervals) |
| ☐ | ☒ | For null hypothesis testing, the test statistic (e.g. $F$, $t$, $r$) with confidence intervals, effect sizes, degrees of freedom and $P$ value noted<br>*Give P values as exact values whenever suitable.* |
| ☒ | ☐ | For Bayesian analysis, information on the choice of priors and Markov chain Monte Carlo settings |
| ☒ | ☐ | For hierarchical and complex designs, identification of the appropriate level for tests and full reporting of outcomes |
| ☐ | ☒ | Estimates of effect sizes (e.g. Cohen's *d*, Pearson's *r*), indicating how they were calculated |

*Our web collection on statistics for biologists contains articles on many of the points above.*

## Software and code

Policy information about availability of computer code

| | |
|---|---|
| Data collection | No software was used. |
| Data analysis | The data analysis was performed by using OriginPro 9 and R v3.6. |

For manuscripts utilizing custom algorithms or software that are central to the research but not yet described in published literature, software must be made available to editors and reviewers. We strongly encourage code deposition in a community repository (e.g. GitHub). See the Nature Portfolio guidelines for submitting code & software for further information.

## Data

Policy information about availability of data

All manuscripts must include a data availability statement. This statement should provide the following information, where applicable:

- Accession codes, unique identifiers, or web links for publicly available datasets
- A description of any restrictions on data availability
- For clinical datasets or third party data, please ensure that the statement adheres to our policy

All relevant data generated during this study are available from the corresponding author on reasonable request.

## Human research participants

Policy information about studies involving human research participants and Sex and Gender in Research.

| | |
|---|---|
| Reporting on sex and gender | We applied sex-adjusted analyses. The geneder variable was not considered in the present study. A detailed overview of sex prevalences in the different clinical groups considered is present in Table 1. |
| Population characteristics | Among patients referred to our Center in the period 2016-2019 we retrospectively selected a consecutive series of 88 patients with Parkinson's disease, 25 patients with mild cognitive impairment due to Alzheimer's disease, and 44 cognitively healthy patients with other neurological diseases, i.e., mononeuropathy, dizziness, headache, for whom cerebrospinal fluid and clinical follow-up at 2 years could be available. |
| Recruitment | The subjects retrospectively selected for the present study were a consecutive series of patients affected by Parkinson's disease (PD) and Azheimer's disease (AD), and cognitively healthy patients with other minor neurological diseases (OND) referring to our Center in the period 2016-2019.<br>The following criteria were considered for PD patients recruitment. Inclusion criteria: diagnosis of clinically established PD, optimal response to dopaminergic challenge test, age at lumbar puncture (LP) between 60 and 80 years old, disease duration since symptoms onset < 5 years, early - moderate disease stage (H&Y< 3), de novo drug-naïve patients or patients treated with low-middle dosage of dopaminergic drugs. Exclusion criteria: L-dopa-induced dyskinesia, motor fluctuations and behavioral disturbances, diagnosis of PD with dementia, diagnosis of atypical parkinsonian syndromes, relevant vascular encephalopathy on brain imaging and recent major stroke, recent traumatic brain injury, major systemic disorders.<br>Inclusion criteria for AD patients were: CSF biomarkers profile (A+/T+), CDR = 0.5, age at LP between 60 and 80 years.<br>Inclusion criteria for OND subjects: minor neurological diseases for which CSF analysis was performed as part of the diagnostic work-up, negative CSF AD core biomarkers, normal neuropsychological evaluation at baseline and at 2-year follow-up, no vascular encephalopathy, recent major stroke, brain injury and major systemic disorders, no ongoing treatments with anti-inflammatory drugs. |
| Ethics oversight | Similar to what done for AD, in our Center CSF analysis is a routine procedure also in PD patients, as approved by our local Ethics Committee CER Umbria (protocol N∘:19369/08/AV, registry N∘: 1287/08, date: 9 October 2008). All patients gave their written inform consent for the study participation. |

Note that full information on the approval of the study protocol must also be provided in the manuscript.

# Field-specific reporting

Please select the one below that is the best fit for your research. If you are not sure, read the appropriate sections before making your selection.

☒ Life sciences ☐ Behavioural & social sciences ☐ Ecological, evolutionary & environmental sciences

For a reference copy of the document with all sections, see nature.com/documents/nr-reporting-summary-flat.pdf

# Life sciences study design

All studies must disclose on these points even when the disclosure is negative.

| | |
|---|---|
| Sample size | This was an explorative study since no other studies attempted at determining a CSF molecular signature of PD-MCI. However, thanks to some preliminary data, we determined a minimum sample size of 25 subjects per group for logitudinal analyses which were needed to determine statistical significant correlations. |
| Data exclusions | No data were excluded. |
| Replication | All the biochemical analyses were performed by means of commercial, commonly used, and highly reliable ELISA kits or Lumipulse G600-II fully automated chemiluminescent enzyme immunoassay system.<br>The analyses were performed using one batch of reagents with intra-assay coefficients of variation below 10%. |
| Randomization | CSF samples were randomized in well-plates for each of the experiments performed. |
| Blinding | All analyses were performed by board-certified laboratory technicians blinded to clinical data. |

# Reporting for specific materials, systems and methods

We require information from authors about some types of materials, experimental systems and methods used in many studies. Here, indicate whether each material, system or method listed is relevant to your study. If you are not sure if a list item applies to your research, read the appropriate section before selecting a response.

## Materials & experimental systems

| n/a | Involved in the study |
|---|---|
| ☐ | ☐ Antibodies |
| ☐ | ☐ Eukaryotic cell lines |
| ☐ | ☐ Palaeontology and archaeology |
| ☐ | ☐ Animals and other organisms |
| ☐ | ☒ Clinical data |
| ☐ | ☐ Dual use research of concern |

## Methods

| n/a | Involved in the study |
|---|---|
| ☐ | ☐ ChIP-seq |
| ☐ | ☐ Flow cytometry |
| ☐ | ☐ MRI-based neuroimaging |

# Antibodies

| | |
|---|---|
| Antibodies used | *Describe all antibodies used in the study; as applicable, provide supplier name, catalog number, clone name, and lot number.* |
| Validation | *Describe the validation of each primary antibody for the species and application, noting any validation statements on the manufacturer's website, relevant citations, antibody profiles in online databases, or data provided in the manuscript.* |

# Eukaryotic cell lines

Policy information about cell lines and Sex and Gender in Research

| | |
|---|---|
| Cell line source(s) | *State the source of each cell line used and the sex of all primary cell lines and cells derived from human participants or vertebrate models.* |
| Authentication | *Describe the authentication procedures for each cell line used OR declare that none of the cell lines used were authenticated.* |
| Mycoplasma contamination | *Confirm that all cell lines tested negative for mycoplasma contamination OR describe the results of the testing for mycoplasma contamination OR declare that the cell lines were not tested for mycoplasma contamination.* |
| Commonly misidentified lines (See ICLAC register) | *Name any commonly misidentified cell lines used in the study and provide a rationale for their use.* |

# Palaeontology and Archaeology

| | |
|---|---|
| Specimen provenance | *Provide provenance information for specimens and describe permits that were obtained for the work (including the name of the issuing authority, the date of issue, and any identifying information). Permits should encompass collection and, where applicable, export.* |
| Specimen deposition | *Indicate where the specimens have been deposited to permit free access by other researchers.* |
| Dating methods | *If new dates are provided, describe how they were obtained (e.g. collection, storage, sample pretreatment and measurement), where they were obtained (i.e. lab name), the calibration program and the protocol for quality assurance OR state that no new dates are provided.* |

☐ Tick this box to confirm that the raw and calibrated dates are available in the paper or in Supplementary Information.

| | |
|---|---|
| Ethics oversight | *Identify the organization(s) that approved or provided guidance on the study protocol, OR state that no ethical approval or guidance was required and explain why not.* |

Note that full information on the approval of the study protocol must also be provided in the manuscript.

# Animals and other research organisms

Policy information about studies involving animals; ARRIVE guidelines recommended for reporting animal research, and Sex and Gender in Research

| | |
|---|---|
| Laboratory animals | *For laboratory animals, report species, strain and age OR state that the study did not involve laboratory animals.* |
| Wild animals | *Provide details on animals observed in or captured in the field; report species and age where possible. Describe how animals were caught and transported and what happened to captive animals after the study (if killed, explain why and describe method; if released, say where and when) OR state that the study did not involve wild animals.* |
| Reporting on sex | *Indicate if findings apply to only one sex; describe whether sex was considered in study design, methods used for assigning sex. Provide data disaggregated for sex where this information has been collected in the source data as appropriate; provide overall numbers in this Reporting Summary. Please state if this information has not been collected. Report sex-based analyses where performed, justify reasons for lack of sex-based analysis.* |

| | |
|---|---|
| Field-collected samples | *For laboratory work with field-collected samples, describe all relevant parameters such as housing, maintenance, temperature, photoperiod and end-of-experiment protocol OR state that the study did not involve samples collected from the field.* |
| Ethics oversight | *Identify the organization(s) that approved or provided guidance on the study protocol, OR state that no ethical approval or guidance was required and explain why not.* |

Note that full information on the approval of the study protocol must also be provided in the manuscript.

## Clinical data

Policy information about clinical studies

All manuscripts should comply with the ICMJE guidelines for publication of clinical research and a completed CONSORT checklist must be included with all submissions.

| | |
|---|---|
| Clinical trial registration | *Provide the trial registration number from ClinicalTrials.gov or an equivalent agency.* |
| Study protocol | 19369/08/AV |
| Data collection | Among patients referred to our Center in the period 2016-2019 we retrospectively selected a consecutive series of 88 PD patients, 25 MCI-AD patients and 44 cognitively healthy patients with OND, i.e., mononeuropathy, dizziness, headache, for whom CSF and clinical follow-up at 2 years could be available. Similar to what done for Alzheimer's disease, in our Center CSF analysis is a routine procedure also in PD patients, as approved by our local Ethics Committee (protocol N∘:19369/08/AV, registry N∘: 1287/08, date: 9 October 2008).<br>At baseline, PD patients underwent a thorough clinical evaluation including motor assessment and neuropsychological testing, as well as brain imaging to rule out other causes. Motor impairment and disease-related disability were assessed by means of UPDRS-III and H&Y, respectively. Neuropsychological evaluation included MMSE and MoCA as cognitive screening tests, and a comprehensive neuropsychological battery exploring attention and working memory, executive functions, memory, language and visuospatial functions. Based on neuropsychological evaluation, patients with no cognitive impairment were defined as PD-CN, while PD-MCI patients were defined according to MDS Task Force criteria, level II. After 2 years, cognitive worsening was defined as a loss of at least 2 points in both MMSE and MoCA, based on available evidence from previous literature.<br>Lumbar puncture was performed according to international guidelines. Briefly, 10–12 mL of CSF were collected in sterile polypropylene tubes and centrifuged at room temperature for 10 min (2000×g). Aliquots (0.5 mL) were frozen at −80 ∘C. All the analyses were carried out by means of commercially available kits. Aβ40, Aβ42, t-tau and p-tau were analyzed using Lumipulse G600-II fully automated chemiluminescent enzyme immunoassay system (Fujirebio Europe, Gent, Belgium). NfL concentrations were determined with NF-light (UmanDiagnostics, Umeå, Sweden), p-NfH with p-NfH (Euroimmun, Lubeck, Germany), Ng with Neurogranin (Trunc P75) (Euroimmun, Lubeck, Germany), t-α-syn with Alpha-Synuclein (Euroimmun, Lubeck, Germany), sAPPα with sAPP-alpha (Tecan, Männedorf, Switzerland), sAPPβ with sAPP-beta wild type (Tecan, Männedorf, Switzerland), sTREM-2 with Human TREM2 (Abcam, Cambridge, United Kingdom), and YKL-40 with Human Chitinase 3-like 1 Quantikine (R&D Systems, Minneapolis, MN). All analyses were performed by board-certified laboratory technicians blinded to clinical data. The analyses were performed using one batch of reagents with intra-assay coefficients of variation below 10%. |
| Outcomes | The aims of the present study were: i) to investigate whether a well-characterized cohort of PD-MCI patients, compared to PD-CN individuals, show a differential neurochemical profile by measuring a large panel of CSF putative biomarkers reflecting different pathophysiological pathways, ii) to verify changes in cognitive performances after 2 years, also investigating the potential predictive role of CSF biomarkers on cognitive outcome.<br>To address such aims: - Wilcoxon-Mann-Whitney sum rank test was used to compare biomarkers distributions among groups (all p-values were corrected for multiple group comparisons), - correlations among CSF biomarkers, and among CSF biomarkers and clinical measures were calculated by means of Spearman's (ρ) correlation coefficient, - linear regression was applied to further assess the significance of the differences observed among PD groups and the significance of the associations between biomarker levels and clinical parameters by assuming age, sex and disease duration as covariates, - logistic regression was applied to determine differences in CSF biomarkers levels between PD-MCI with and without cognitive worsening assuming age, sex and disease duration as covariates. |

## Dual use research of concern

Policy information about dual use research of concern

### Hazards

Could the accidental, deliberate or reckless misuse of agents or technologies generated in the work, or the application of information presented in the manuscript, pose a threat to:

| No | Yes | |
|---|---|---|
| ☐ | ☐ | Public health |
| ☐ | ☐ | National security |
| ☐ | ☐ | Crops and/or livestock |
| ☐ | ☐ | Ecosystems |
| ☐ | ☐ | Any other significant area |

### Experiments of concern

Does the work involve any of these experiments of concern:

| No | Yes | |
|---|---|---|
| ☐ | ☐ | Demonstrate how to render a vaccine ineffective |
| ☐ | ☐ | Confer resistance to therapeutically useful antibiotics or antiviral agents |
| ☐ | ☐ | Enhance the virulence of a pathogen or render a nonpathogen virulent |
| ☐ | ☐ | Increase transmissibility of a pathogen |
| ☐ | ☐ | Alter the host range of a pathogen |
| ☐ | ☐ | Enable evasion of diagnostic/detection modalities |
| ☐ | ☐ | Enable the weaponization of a biological agent or toxin |
| ☐ | ☐ | Any other potentially harmful combination of experiments and agents |

## ChIP-seq

### Data deposition

☐ Confirm that both raw and final processed data have been deposited in a public database such as GEO.

☐ Confirm that you have deposited or provided access to graph files (e.g. BED files) for the called peaks.

| | |
|---|---|
| Data access links<br>*May remain private before publication.* | *For "Initial submission" or "Revised version" documents, provide reviewer access links. For your "Final submission" document, provide a link to the deposited data.* |
| Files in database submission | *Provide a list of all files available in the database submission.* |
| Genome browser session<br>(e.g. UCSC) | *Provide a link to an anonymized genome browser session for "Initial submission" and "Revised version" documents only, to enable peer review. Write "no longer applicable" for "Final submission" documents.* |

### Methodology

| | |
|---|---|
| Replicates | *Describe the experimental replicates, specifying number, type and replicate agreement.* |
| Sequencing depth | *Describe the sequencing depth for each experiment, providing the total number of reads, uniquely mapped reads, length of reads and whether they were paired- or single-end.* |
| Antibodies | *Describe the antibodies used for the ChIP-seq experiments; as applicable, provide supplier name, catalog number, clone name, and lot number.* |
| Peak calling parameters | *Specify the command line program and parameters used for read mapping and peak calling, including the ChIP, control and index files used.* |
| Data quality | *Describe the methods used to ensure data quality in full detail, including how many peaks are at FDR 5% and above 5-fold enrichment.* |
| Software | *Describe the software used to collect and analyze the ChIP-seq data. For custom code that has been deposited into a community repository, provide accession details.* |

## Flow Cytometry

### Plots

Confirm that:

☐ The axis labels state the marker and fluorochrome used (e.g. CD4-FITC).

☐ The axis scales are clearly visible. Include numbers along axes only for bottom left plot of group (a 'group' is an analysis of identical markers).

☐ All plots are contour plots with outliers or pseudocolor plots.

☐ A numerical value for number of cells or percentage (with statistics) is provided.

### Methodology

| | |
|---|---|
| Sample preparation | *Describe the sample preparation, detailing the biological source of the cells and any tissue processing steps used.* |
| Instrument | *Identify the instrument used for data collection, specifying make and model number.* |

Software | *Describe the software used to collect and analyze the flow cytometry data. For custom code that has been deposited into a community repository, provide accession details.*

Cell population abundance | *Describe the abundance of the relevant cell populations within post-sort fractions, providing details on the purity of the samples and how it was determined.*

Gating strategy | *Describe the gating strategy used for all relevant experiments, specifying the preliminary FSC/SSC gates of the starting cell population, indicating where boundaries between "positive" and "negative" staining cell populations are defined.*

☐ Tick this box to confirm that a figure exemplifying the gating strategy is provided in the Supplementary Information.

# Magnetic resonance imaging

## Experimental design

Design type | *Indicate task or resting state; event-related or block design.*

Design specifications | *Specify the number of blocks, trials or experimental units per session and/or subject, and specify the length of each trial or block (if trials are blocked) and interval between trials.*

Behavioral performance measures | *State number and/or type of variables recorded (e.g. correct button press, response time) and what statistics were used to establish that the subjects were performing the task as expected (e.g. mean, range, and/or standard deviation across subjects).*

## Acquisition

Imaging type(s) | *Specify: functional, structural, diffusion, perfusion.*

Field strength | *Specify in Tesla*

Sequence & imaging parameters | *Specify the pulse sequence type (gradient echo, spin echo, etc.), imaging type (EPI, spiral, etc.), field of view, matrix size, slice thickness, orientation and TE/TR/flip angle.*

Area of acquisition | *State whether a whole brain scan was used OR define the area of acquisition, describing how the region was determined.*

Diffusion MRI ☐ Used ☐ Not used

## Preprocessing

Preprocessing software | *Provide detail on software version and revision number and on specific parameters (model/functions, brain extraction, segmentation, smoothing kernel size, etc.).*

Normalization | *If data were normalized/standardized, describe the approach(es): specify linear or non-linear and define image types used for transformation OR indicate that data were not normalized and explain rationale for lack of normalization.*

Normalization template | *Describe the template used for normalization/transformation, specifying subject space or group standardized space (e.g. original Talairach, MNI305, ICBM152) OR indicate that the data were not normalized.*

Noise and artifact removal | *Describe your procedure(s) for artifact and structured noise removal, specifying motion parameters, tissue signals and physiological signals (heart rate, respiration).*

Volume censoring | *Define your software and/or method and criteria for volume censoring, and state the extent of such censoring.*

## Statistical modeling & inference

Model type and settings | *Specify type (mass univariate, multivariate, RSA, predictive, etc.) and describe essential details of the model at the first and second levels (e.g. fixed, random or mixed effects; drift or auto-correlation).*

Effect(s) tested | *Define precise effect in terms of the task or stimulus conditions instead of psychological concepts and indicate whether ANOVA or factorial designs were used.*

Specify type of analysis: ☐ Whole brain ☐ ROI-based ☐ Both

Statistic type for inference
(See Eklund et al. 2016) | *Specify voxel-wise or cluster-wise and report all relevant parameters for cluster-wise methods.*

Correction | *Describe the type of correction and how it is obtained for multiple comparisons (e.g. FWE, FDR, permutation or Monte Carlo).*

## Models & analysis

| n/a | Involved in the study |
| --- | --- |
| ☐ | ☐ Functional and/or effective connectivity |
| ☐ | ☐ Graph analysis |
| ☐ | ☐ Multivariate modeling or predictive analysis |

Functional and/or effective connectivity

*Report the measures of dependence used and the model details (e.g. Pearson correlation, partial correlation, mutual information).*

Graph analysis

*Report the dependent variable and connectivity measure, specifying weighted graph or binarized graph, subject- or group-level, and the global and/or node summaries used (e.g. clustering coefficient, efficiency, etc.).*

Multivariate modeling and predictive analysis

*Specify independent variables, features extraction and dimension reduction, model, training and evaluation metrics.*
